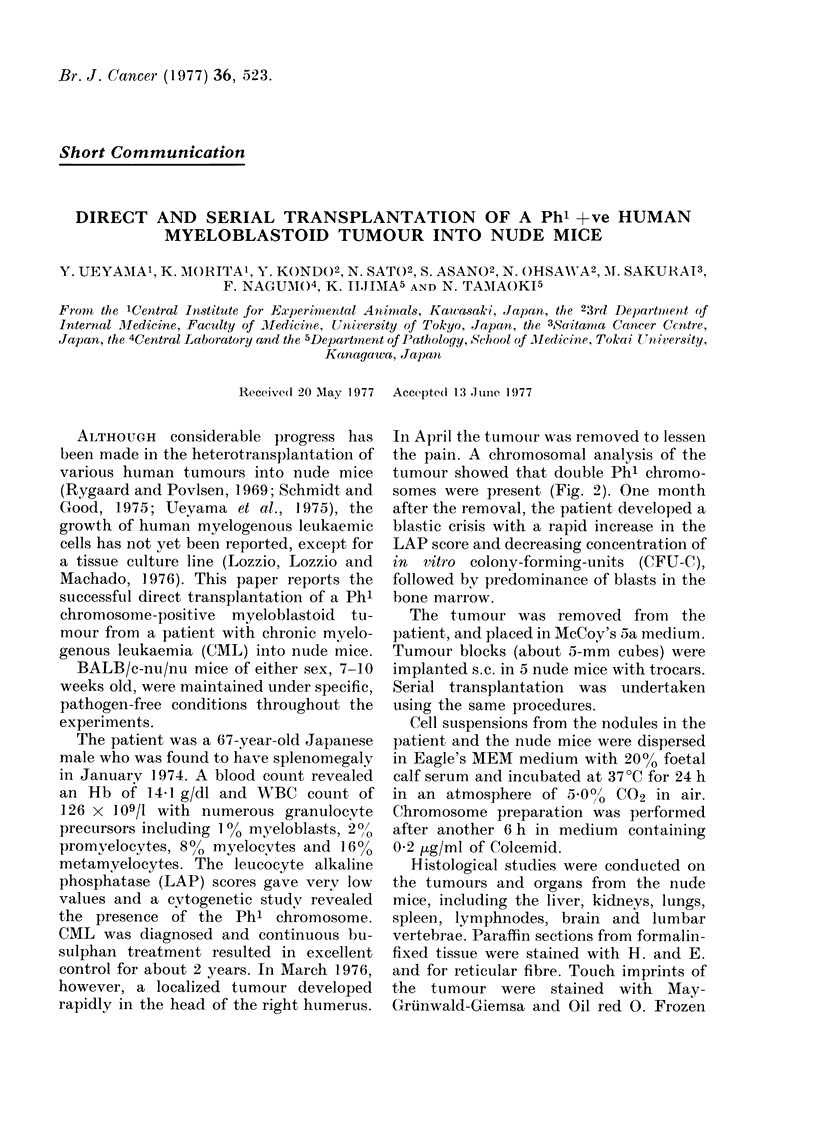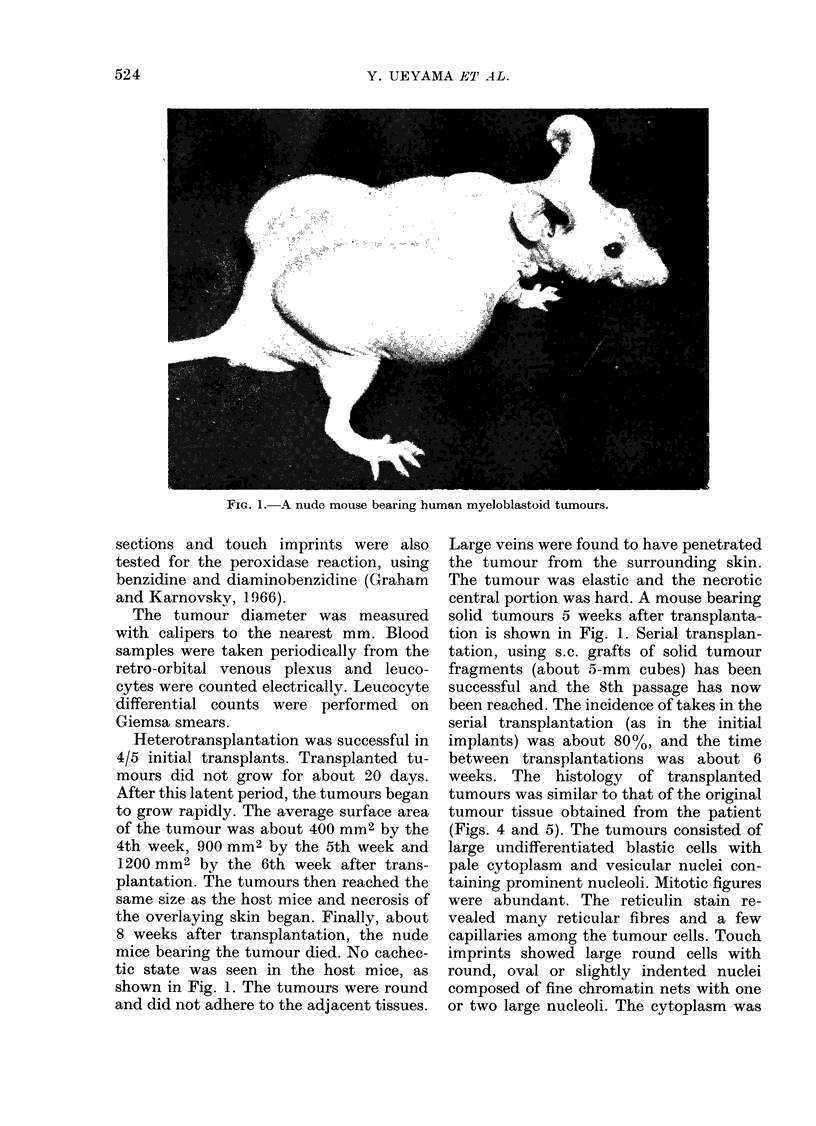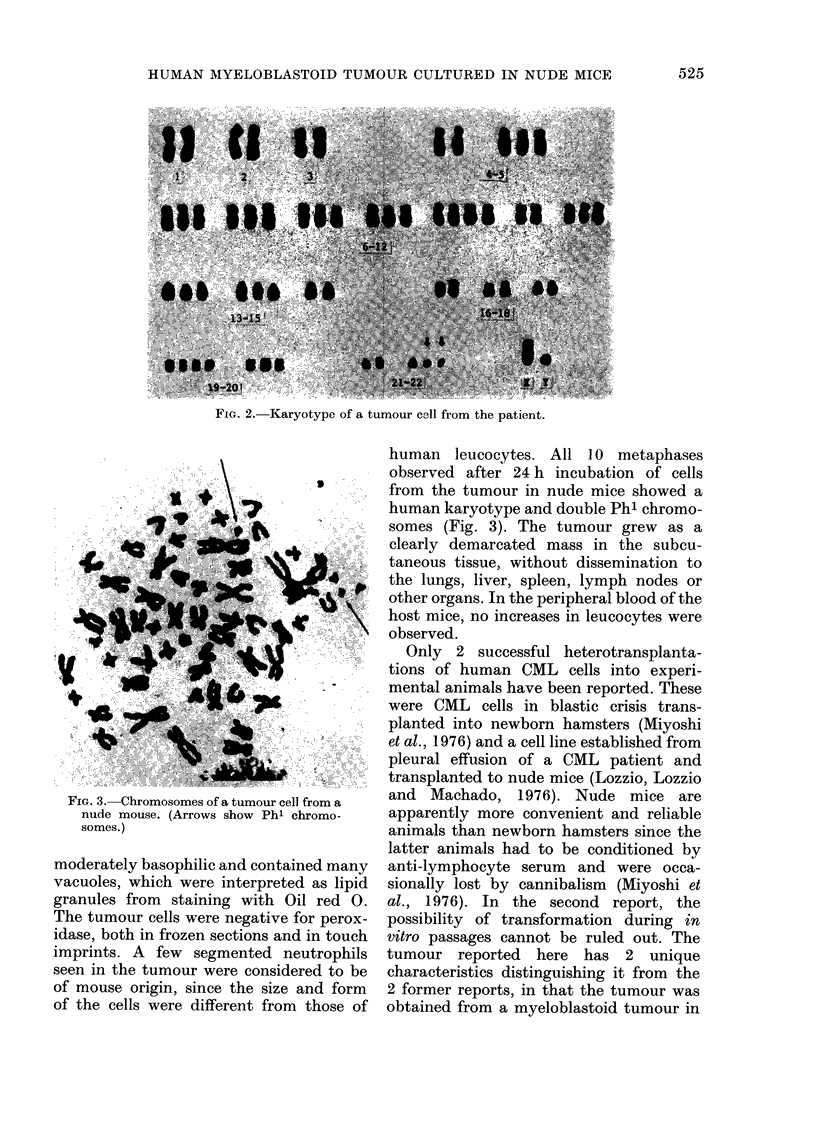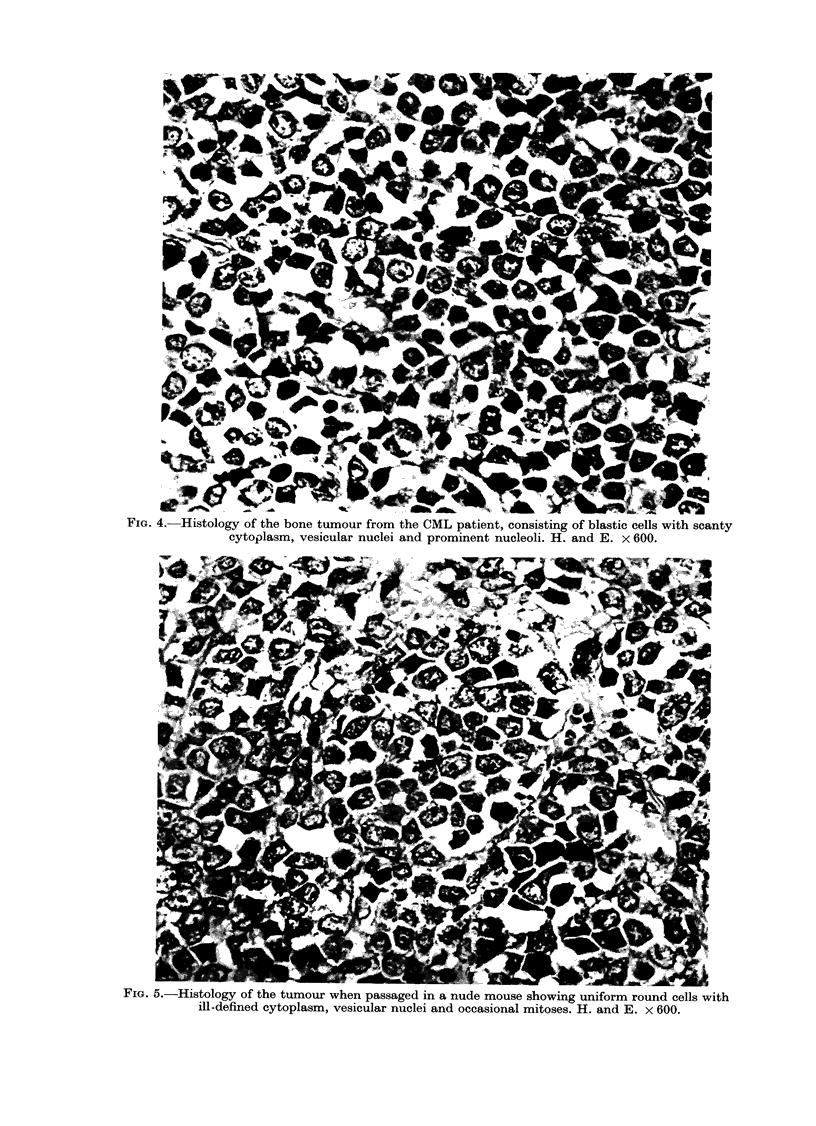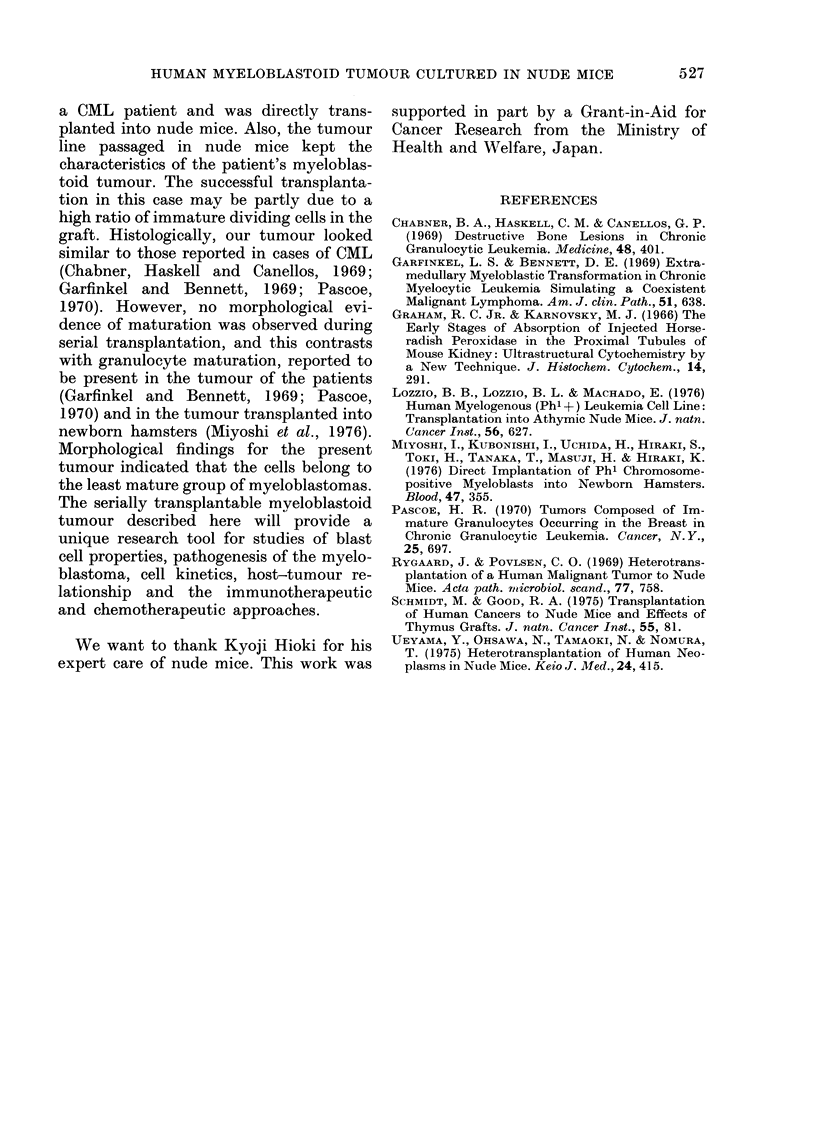# Direct and serial transplantation of a Ph + ve human myeloblastoid tumour into nude mice.

**DOI:** 10.1038/bjc.1977.222

**Published:** 1977-10

**Authors:** Y. Ueyama, K. Morita, Y. Kondo, N. Sato, S. Asano, N. Ohsawa, M. Sakurai, F. Nagumo, K. Iijima, N. Tamaoki

## Abstract

**Images:**


*Br. J. Cancer* (1977) **36**, 523.

***Short Communication***

### DIRECT AND SERIAL TRANSPLANTATION OF A $Ph^1$ +ve HUMAN MYELOBLASTOID TUMOUR INTO NUDE MICE

Y. UEYAMA[1], K. MORITA[1], Y. KONDO[2], N. SATO[2], S. ASANO[2], N. OHSAWA[2], M. SAKURAI[3], F. NAGUMO[4], K. IIJIMA[5] AND N. TAMAOKI[5]

*From the [1]Central Institute for Experimental Animals, Kawasaki, Japan, the [2]3rd Department of Internal Medicine, Faculty of Medicine, University of Tokyo, Japan, the [3]Saitama Cancer Centre, Japan, the [4]Central Laboratory and the [5]Department of Pathology, School of Medicine, Tokai University, Kanagawa, Japan*

Received 20 May 1977 Accepted 13 June 1977

ALTHOUGH considerable progress has been made in the heterotransplantation of various human tumours into nude mice (Rygaard and Povlsen, 1969; Schmidt and Good, 1975; Ueyama *et al.*, 1975), the growth of human myelogenous leukaemic cells has not yet been reported, except for a tissue culture line (Lozzio, Lozzio and Machado, 1976). This paper reports the successful direct transplantation of a $Ph^1$ chromosome-positive myeloblastoid tumour from a patient with chronic myelogenous leukaemia (CML) into nude mice.

BALB/c-nu/nu mice of either sex, 7–10 weeks old, were maintained under specific, pathogen-free conditions throughout the experiments.

The patient was a 67-year-old Japanese male who was found to have splenomegaly in January 1974. A blood count revealed an Hb of 14·1 g/dl and WBC count of $126 \times 10^9$/l with numerous granulocyte precursors including 1% myeloblasts, 2% promyelocytes, 8% myelocytes and 16% metamyelocytes. The leucocyte alkaline phosphatase (LAP) scores gave very low values and a cytogenetic study revealed the presence of the $Ph^1$ chromosome. CML was diagnosed and continuous busulphan treatment resulted in excellent control for about 2 years. In March 1976, however, a localized tumour developed rapidly in the head of the right humerus. In April the tumour was removed to lessen the pain. A chromosomal analysis of the tumour showed that double $Ph^1$ chromosomes were present (Fig. 2). One month after the removal, the patient developed a blastic crisis with a rapid increase in the LAP score and decreasing concentration of *in vitro* colony-forming-units (CFU-C), followed by predominance of blasts in the bone marrow.

The tumour was removed from the patient, and placed in McCoy's 5a medium. Tumour blocks (about 5-mm cubes) were implanted s.c. in 5 nude mice with trocars. Serial transplantation was undertaken using the same procedures.

Cell suspensions from the nodules in the patient and the nude mice were dispersed in Eagle's MEM medium with 20% foetal calf serum and incubated at 37°C for 24 h in an atmosphere of 5·0% $CO_2$ in air. Chromosome preparation was performed after another 6 h in medium containing 0·2 μg/ml of Colcemid.

Histological studies were conducted on the tumours and organs from the nude mice, including the liver, kidneys, lungs, spleen, lymphnodes, brain and lumbar vertebrae. Paraffin sections from formalin-fixed tissue were stained with H. and E. and for reticular fibre. Touch imprints of the tumour were stained with May-Grünwald-Giemsa and Oil red O. Frozen

FIG. 1.—A nude mouse bearing human myeloblastoid tumours.

sections and touch imprints were also tested for the peroxidase reaction, using benzidine and diaminobenzidine (Graham and Karnovsky, 1966).

The tumour diameter was measured with calipers to the nearest mm. Blood samples were taken periodically from the retro-orbital venous plexus and leucocytes were counted electrically. Leucocyte differential counts were performed on Giemsa smears.

Heterotransplantation was successful in 4/5 initial transplants. Transplanted tumours did not grow for about 20 days. After this latent period, the tumours began to grow rapidly. The average surface area of the tumour was about 400 $mm^2$ by the 4th week, 900 $mm^2$ by the 5th week and 1200 $mm^2$ by the 6th week after transplantation. The tumours then reached the same size as the host mice and necrosis of the overlaying skin began. Finally, about 8 weeks after transplantation, the nude mice bearing the tumour died. No cachectic state was seen in the host mice, as shown in Fig. 1. The tumours were round and did not adhere to the adjacent tissues. Large veins were found to have penetrated the tumour from the surrounding skin. The tumour was elastic and the necrotic central portion was hard. A mouse bearing solid tumours 5 weeks after transplantation is shown in Fig. 1. Serial transplantation, using s.c. grafts of solid tumour fragments (about 5-mm cubes) has been successful and the 8th passage has now been reached. The incidence of takes in the serial transplantation (as in the initial implants) was about 80%, and the time between transplantations was about 6 weeks. The histology of transplanted tumours was similar to that of the original tumour tissue obtained from the patient (Figs. 4 and 5). The tumours consisted of large undifferentiated blastic cells with pale cytoplasm and vesicular nuclei containing prominent nucleoli. Mitotic figures were abundant. The reticulin stain revealed many reticular fibres and a few capillaries among the tumour cells. Touch imprints showed large round cells with round, oval or slightly indented nuclei composed of fine chromatin nets with one or two large nucleoli. The cytoplasm was

FIG. 2.—Karyotype of a tumour cell from the patient.

FIG. 3.—Chromosomes of a tumour cell from a nude mouse. (Arrows show Ph[1] chromosomes.)

moderately basophilic and contained many vacuoles, which were interpreted as lipid granules from staining with Oil red O. The tumour cells were negative for peroxidase, both in frozen sections and in touch imprints. A few segmented neutrophils seen in the tumour were considered to be of mouse origin, since the size and form of the cells were different from those of human leucocytes. All 10 metaphases observed after 24 h incubation of cells from the tumour in nude mice showed a human karyotype and double Ph[1] chromosomes (Fig. 3). The tumour grew as a clearly demarcated mass in the subcutaneous tissue, without dissemination to the lungs, liver, spleen, lymph nodes or other organs. In the peripheral blood of the host mice, no increases in leucocytes were observed.

Only 2 successful heterotransplantations of human CML cells into experimental animals have been reported. These were CML cells in blastic crisis transplanted into newborn hamsters (Miyoshi *et al.*, 1976) and a cell line established from pleural effusion of a CML patient and transplanted to nude mice (Lozzio, Lozzio and Machado, 1976). Nude mice are apparently more convenient and reliable animals than newborn hamsters since the latter animals had to be conditioned by anti-lymphocyte serum and were occasionally lost by cannibalism (Miyoshi *et al.*, 1976). In the second report, the possibility of transformation during *in vitro* passages cannot be ruled out. The tumour reported here has 2 unique characteristics distinguishing it from the 2 former reports, in that the tumour was obtained from a myeloblastoid tumour in

FIG. 4.—Histology of the bone tumour from the CML patient, consisting of blastic cells with scanty cytoplasm, vesicular nuclei and prominent nucleoli. H. and E. $\times 600$.

FIG. 5.—Histology of the tumour when passaged in a nude mouse showing uniform round cells with ill-defined cytoplasm, vesicular nuclei and occasional mitoses. H. and E. $\times 600$.

a CML patient and was directly transplanted into nude mice. Also, the tumour line passaged in nude mice kept the characteristics of the patient's myeloblastoid tumour. The successful transplantation in this case may be partly due to a high ratio of immature dividing cells in the graft. Histologically, our tumour looked similar to those reported in cases of CML (Chabner, Haskell and Canellos, 1969; Garfinkel and Bennett, 1969; Pascoe, 1970). However, no morphological evidence of maturation was observed during serial transplantation, and this contrasts with granulocyte maturation, reported to be present in the tumour of the patients (Garfinkel and Bennett, 1969; Pascoe, 1970) and in the tumour transplanted into newborn hamsters (Miyoshi *et al.*, 1976). Morphological findings for the present tumour indicated that the cells belong to the least mature group of myeloblastomas. The serially transplantable myeloblastoid tumour described here will provide a unique research tool for studies of blast cell properties, pathogenesis of the myeloblastoma, cell kinetics, host–tumour relationship and the immunotherapeutic and chemotherapeutic approaches.

We want to thank Kyoji Hioki for his expert care of nude mice. This work was supported in part by a Grant-in-Aid for Cancer Research from the Ministry of Health and Welfare, Japan.

### REFERENCES

CHABNER, B. A., HASKELL, C. M. & CANELLOS, G. P. (1969) Destructive Bone Lesions in Chronic Granulocytic Leukemia. *Medicine*, **48**, 401.

GARFINKEL, L. S. & BENNETT, D. E. (1969) Extramedullary Myeloblastic Transformation in Chronic Myelocytic Leukemia Simulating a Coexistent Malignant Lymphoma. *Am. J. clin. Path.*, **51**, 638.

GRAHAM, R. C. JR. & KARNOVSKY, M. J. (1966) The Early Stages of Absorption of Injected Horseradish Peroxidase in the Proximal Tubules of Mouse Kidney: Ultrastructural Cytochemistry by a New Technique. *J. Histochem. Cytochem.*, **14**, 291.

LOZZIO, B. B., LOZZIO, B. L. & MACHADO, E. (1976) Human Myelogenous ($Ph^1+$) Leukemia Cell Line: Transplantation into Athymic Nude Mice. *J. natn. Cancer Inst.*, **56**, 627.

MIYOSHI, I., KUBONISHI, I., UCHIDA, H., HIRAKI, S., TOKI, H., TANAKA, T., MASUJI, H. & HIRAKI, K. (1976) Direct Implantation of $Ph^1$ Chromosome-positive Myeloblasts into Newborn Hamsters. *Blood*, **47**, 355.

PASCOE, H. R. (1970) Tumors Composed of Immature Granulocytes Occurring in the Breast in Chronic Granulocytic Leukemia. *Cancer, N.Y.*, **25**, 697.

RYGAARD, J. & POVLSEN, C. O. (1969) Heterotransplantation of a Human Malignant Tumor to Nude Mice. *Acta path. microbiol. scand.*, **77**, 758.

SCHMIDT, M. & GOOD, R. A. (1975) Transplantation of Human Cancers to Nude Mice and Effects of Thymus Grafts. *J. natn. Cancer Inst.*, **55**, 81.

UEYAMA, Y., OHSAWA, N., TAMAOKI, N. & NOMURA, T. (1975) Heterotransplantation of Human Neoplasms in Nude Mice. *Keio J. Med.*, **24**, 415.